# Circadian Tick‐Talking Across the Neuroendocrine System and Suprachiasmatic Nuclei Circuits: The Enigmatic Communication Between the Molecular and Electrical Membrane Clocks

**DOI:** 10.1111/jne.12279

**Published:** 2015-06-22

**Authors:** M. D. C. Belle

**Affiliations:** ^1^ Faculty of Life SciencesUniversity of ManchesterManchesterUK

**Keywords:** suprachiasmatic nuclei, circadian rhythm, clock genes, neuroendocrine system, reproduction, electrical activity, ion channels

## Abstract

As with many processes in nature, appropriate timing in biological systems is of paramount importance. In the neuroendocrine system, the efficacy of hormonal influence on major bodily functions, such as reproduction, metabolism and growth, relies on timely communication within and across many of the brain's homeostatic systems. The activity of these circuits is tightly orchestrated with the animal's internal physiological demands and external solar cycle by a master circadian clock. In mammals, this master clock is located in the hypothalamic suprachiasmatic nucleus (SCN), where the ensemble activity of thousands of clock neurones generates and communicates circadian time cues to the rest of the brain and body. Many regions of the brain, including areas with neuroendocrine function, also contain local daily clocks that can provide feedback signals to the SCN. Although much is known about the molecular processes underpinning endogenous circadian rhythm generation in SCN neurones and, to a lesser extent, extra‐SCN cells, the electrical membrane clock that acts in partnership with the molecular clockwork to communicate circadian timing across the brain is poorly understood. The present review focuses on some circadian aspects of reproductive neuroendocrinology and processes involved in circadian rhythm communication in the SCN, aiming to identify key gaps in our knowledge of cross‐talk between our daily master clock and neuroendocrine function. The intention is to highlight our surprisingly limited understanding of their interaction in the hope that this will stimulate future work in these areas.

The developmental onset of some fundamental neuroendocrine processes of the body, such as those involved in reproductive maturity, ranges from months to years depending on the species in question, and relies on constellations of tightly regulated hormonal signalling processes within the brain and body. Once established, this timely coordinated balance in hormonal activity and reproduction is temporally regulated by our master circadian clock in the suprachiasmatic nuclei (SCN). Indeed, work spanning over 50 years is unravelling the important and intimate link between SCN activity and appropriate timing in neuroendocrine function.

Circadian rhythms are daily timing in behaviour and physiology that persist (free run) in the absence of external time cues, such as light. In mammals, the master circadian clock resides in the SCN, where the activity of thousands of cell‐autonomous clock neurones is synchronised each day to output ensemble circadian time cues to the rest of the brain and body. At its most basic level, the circadian clockwork is conceptualised as an intrinsic intracellular process involving a dynamic interplay between the protein products of core clock genes such as *Period1/2* (*Per1/2*), *Cryptochrome 1/2* (*Cry 1/2*), *Clock* and *Bmal1*. In the past three decades, significant progress has been made to dissect and understand the intricate inner workings of this molecular timing machine that operates as a near 24‐h transcription–translation molecular feedback loop (TTFL) 1, 2.

Our current understanding of circadian rhythm generation in the SCN is that the TTFL activity within single SCN neurones drives daily changes in membrane excitability 3, 4 (although see also the later section on the membrane clock). Indeed, neurones of the central nervous system can alter their intrinsic excitability by modulating the activity of their ion channels that provide excitatory or inhibitory drive to the membrane 5. How the TTFL achieves this, however, is largely unknown. Broadly speaking, activation of sodium and some calcium channels and/or reduction in potassium channel activity depolarise the resting membrane potential (RMP) of the neurones, causing action potential (AP) discharge. By contrast, activation of potassium and/or chloride channels provides hyperpolarising forces that suppress electrical activity. In SCN neurones, both the conductivity and transcript activity of several ion channels are under circadian control 3, 4. This allows SCN neurones to generate daily rhythms in electrical outputs, a hallmark feature of SCN cells that is paramount to the functioning of the circadian timing system. Measurements of AP firing rate in unidentified SCN neurones both *in vivo* and *in vitro* SCN brain slice preparations show that, at the population level, SCN neurones are significantly more active during the day (an up state: discharging APs at 4–6 Hz) than at night (a down state: generating APs at 0.1–2 Hz) (Fig. 1. Even when isolated from the SCN tissue, single SCN neurones retain the intrinsic ability to generate daily rhythm in AP activity 6, 7 that can be maintained for days. It is considered that this day–night alteration in electrical states synchronises the activity of SCN neurones and communicates circadian information to the brain and body 3. Evidence from imaging and electrophysiological studies, however, suggests that the manner through which SCN neurones encode and communicate circadian information is more complex than first assumed and may not solely rely on day–night variation in AP firing rate.

**Figure 1:**
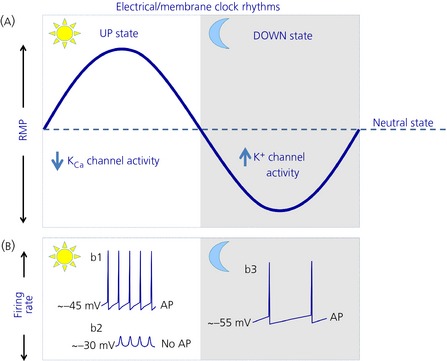
A schematic view of the daily electrical profiles of suprachiasmatic nucleus (SCN) neurones. (a) Over the day–night cycle, SCN neurones show overt changes in their resting membrane potential (RMP), and traverse through several points of neutral rest state (indicated by where the solid line crosses the dashed line). During the day, the RMP of SCN neurones is depolarised. In some cells, reduced activity of L‐type calcium and calcium activated potassium (K_C__a_) channels partly underpins this up state 128. At night, increased conductivity of multiple potassium channels hyperpolarises the neurones, placing them into a down state 3, 4. (b) In some SCN neurones, this daytime up state causes action potential (AP) discharge (b1). In others, however, the RMP becomes too positive (~ −30 mV) to sustain AP production (b2). Instead, these neurones display depolarised low‐amplitude membrane oscillation (b2). At night, during the RMP down state, SCN neurones generate action potentials at a significantly reduced rate (b3). This shows the complexity, richness and diversity of electrical communication in SCN neurones. The light area indicates the day, whereas the shaded region shows the night. The increase or decrease underlying ion channel activity is indicated by upward‐ and downward‐pointing blue arrows, respectively.

Under natural conditions, SCN clock neurones are entrained/synchronised to the light and dark cycle by responding to the daily glutamatergic activity of the retino‐hypothalamic tract (Fig. 2). This excitatory drive is conveyed to the SCN by specialised melanopsin‐containing retinal ganglion cells that communicate ambient light levels directly to SCN neurones 8, causing *Per1* gene activation in these cells 9. This allows environmental light cues to reset SCN clock neurones and coordinate their activity to generate a stable internal representation of solar time.

**Figure 2:**
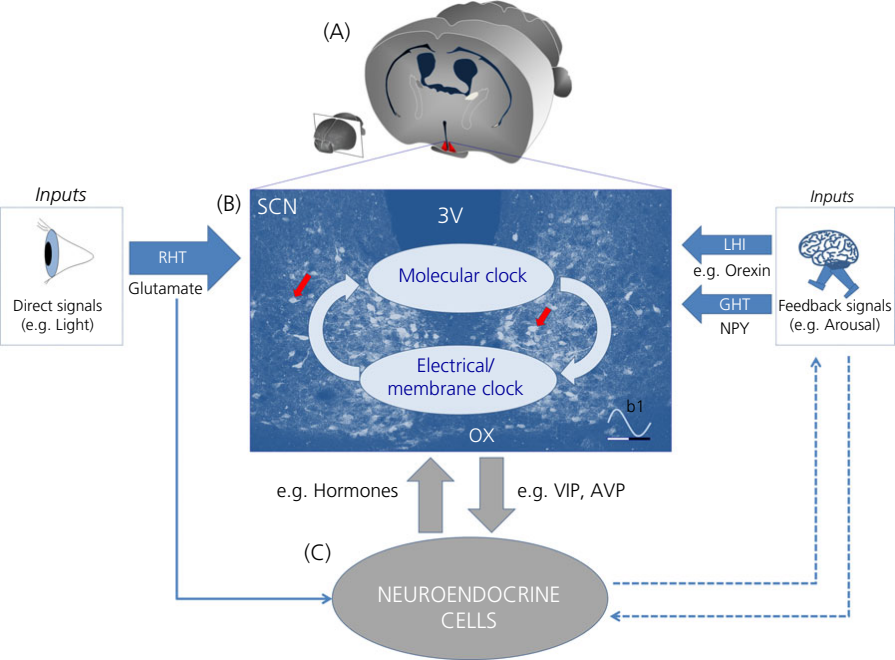
Cross‐talk between the suprachiasmatic nucleus (SCN) and neuroendocrine cells. (a) Cross‐section of the mouse brain at the anatomical level of the SCN (in red). (b) Bidirectional communication between the molecular clock (transcription–translation molecular feedback loop; TTFL) and electrical/membrane clock. In this model, the TTFL clock drives day–night rhythms in the electrical activity of SCN neurones, and electrical activity feedback onto the TTFL clock through unknown mechanisms (light blue arrows). This may underlie how the TTFL outputs signals to neuroendocrine cells (c) and how neuroendocrine processes feedback to adjust circadian timing in the SCN (grey arrows). This drawing is superimposed on top of a modified image taken at the SCN mid‐coronal section showing *Per1*‐EGFP neurones (red arrows). Darker blue arrows represent inputs to the SCN, with their sizes denoting feedback magnitude. Solid arrows indicate an established link, whereas broken arrows show tentative interactions. (b1) Stylised waveform showing daily variation in SCN *Per1* and electrical activity. White and black bars underneath represent day and night, respectively. RHT, retino‐hypothalamic tract; LHI, lateral hypothalamic input; GHT, geniculo‐hypothalamic tract; OX, optic chiasm; 3V, third ventricle; NPY, neuropeptide Y; VIP, vasoactive intestinal polypeptide; AVP, arginine vasopressin.

Several internal physiological signals also feedback and fine tune timing precision in the SCN. Such intrinsic feedback cues [referred to as Zeitnehmer (‘time‐taker’), a term applied to input pathways that are rhythmically regulated by feedback from an oscillator] 10, 11 emerge mainly from the body's homeostatic systems and are communicated to the SCN using a variety of signalling neuropeptides to mainly suppress *Per1/2* gene expression and electrical activity. These feedback signals include neuropeptide Y (NPY) neurones of the thalamic intergeniculate leaflet, which send monosynaptic projections to the SCN via the geniculo‐hypothalamic tract 12, as well as arousal‐promoting orexinergic neurones of the lateral hypothalamus 13, which also send axonal contacts to SCN neurones 14. In the SCN, these neuropeptidergic signals can converge onto single clock neurones to modulate electrical activity and circadian timing in this brain structure 14, 15. As such, SCN neurones integrate both intrinsic and extrinsic signals and send collective circadian time cues by neural and paracrine pathways to the rest of the brain, including many neuroendocrine hypothalamic structures (Fig. 2. Thus, the physiology and behaviour of the organism are tuned to anticipate and adapt to the solar day–night cycle, a key physiological prerequisite for survival and reproductive success. The SCN therefore represents a good example of localised autonomous function in the brain, and provides a unique opportunity in neuroscience to link highly organised behaviours (e.g. sleep–wake cycle, daily feeding drive and neuroendocrine functions) to the activities of a known population of neurones in the brain.

## Circadian regulation of neuroendocrine functions

The realisation of the importance of circadian timing in hormone function started with the work of Everett and Sawyer in the 1950s. In a pioneering study, it was discovered that, in nocturnal rodents, a stimulatory drive occurring at a narrow temporal window in the pro‐oestrus afternoon is necessary for the induction of ovulation later at night 16. Subsequently, a number of studies both in primates (including humans) and rodents have reported circadian rhythms in gonadotrophin‐releasing hormone (GnRH) gene expression 17 and in the levels of many of the body's endocrine hormones, such as luteinising hormone (LH), testosterone, prolactin (PRL), cortisol and gonadotrophins after the onset of puberty 18, 19, 20, 21, 22. A more in‐depth description of hormonal secretion profiles is provided elsewhere 23.

Indeed, when placed in constant conditions, the oestrus cycle in rodents free‐runs 24, 25; a description of circadian terms is provided in Liu *et al*. 26. This supports its reliance on a master timing process governing daily cycles in mammalian endocrine physiology. Furthermore, ablation of the SCN or its output pathways abolishes the night‐time LH surge and circadian rhythms in the release of a number of endocrine hormones, such as PRL and gonadotrophin 27, 28, 29.

Perhaps the most direct evidence of an SCN‐dependent driven rhythm in hormonal secretion comes from elegant studies in female hamsters. Under normal conditions, both halves of the bilateral SCN in hamsters operate in synchrony. When these animals are housed in constant light, however, the SCN activity and behavioural rhythms ‘split’ in some hamsters, giving rise to two activity bouts in a 24‐h period 30. Remarkably, these ‘split’ females display two daily LH surges, each in anti‐phase and of half the concentration seen in ‘nonsplit’ controls 31. More recently, work in rodents has definitively linked circadian clock gene activity in the SCN with circadian timing in hormone synthesis and secretion 32.

Taken together, these studies demonstrate that important aspects of neuroendocrine physiology exhibit circadian variation, and that the master circadian clock is central to the timing of endocrine processes. This provides an excellent model system in which to investigate circadian and endocrine interactions.

## Circadian signalling to neuroendocrine targets

Neurones of the SCN are neurochemically and functionally heterogeneous and form distinct anatomical clusters within this brain structure. Ventral SCN neurones synthesise vasoactive intestinal polypeptide (VIP), whereas the dorsal neurones contain arginine vasopressin (AVP) 33, two neuropeptides that are rhythmically produced in the SCN and are critically involved in appropriate circadian function 6, 34, 35, 36. Although the VPAC_2_ receptor, the preferred receptor for VIP in the SCN, is expressed throughout this nucleus 37, VIP neurones generally project dorsally to the vicinity of AVP cells 33. Here, they form an ensemble bundle with AVP‐axons that project away from the SCN.

In addition to forming conventional cell‐to‐cell synaptic contacts, SCN neurones can also signal circadian timing to the body in a paracrine fashion. Indeed, the timing of behavioural rhythms in rodents, such as locomotor activity, drinking and gnawing, are under the control of the SCN paracrine tone 38, 39, 40, 41. An ingenious *in vitro* study has also recently shown that some intrinsic SCN clock function relies on paracrine communication amongst its neurones (mainly through diffusible neuropeptides such as VIP) 42, although further *in vivo* studies are needed to establish the role of paracrine signalling in SCN function. When sending circadian signals to the neuroendocrine system, however, the SCN does so via direct synaptic contacts onto endocrine cells or through populations of neurones that contact endocrine cells. This suggests that, unlike daily timing in behaviour (neuromodulation) 43, SCN communication to neuroendocrine cells requires the precision and speed of discrete synaptic transmission.

In the hypothalamic preoptic area (POA) of many species, VIP terminals originating from the SCN form direct contacts onto GnRH neurones, and indirectly connect to oestrodiol‐concentrating interneurones that communicate to GnRH cells 30, 44, 45, 46, 47. The VPAC_2_ receptor is expressed by a subset of GnRH neurones 48, 49 and VIP can directly modulate the activity of GnRH cells in a time‐of‐day related manner, therefore controlling the timing of LH release 50, 51, 52, 53. Alteration of VIP signalling both *in vivo* and *in vitro* causes marked changes in GnRH, LH and gonadotrophin release 54, 55, 56, as well as in the magnitude and timing of the LH surge 55, 57. Evidence also suggests that SCN VIP signalling to neuroendocrine dopaminergic neurones is involved in modulating pituitary PRL release 58. Indeed, paraventricular nucleus (PVN) dopaminergic neurones express the VPAC_2_ receptor, hypothalamic VIP expression is in phase with PRL release, and work in rodents indicates that the activity of PVN dopaminergic cells suppresses pituitary PRL secretion 59, 60, 61.

There is also strong evidence in rodents suggesting that AVP signalling in the POA modulates the activity of GnRH neurones and is involved in PRL and LH secretion. AVP fibres contact GnRH neurones, and AVP secretion occurs in phase with GnRH release 45, 62. Furthermore, work in female rats shows that mRNA for the AVP receptor (*V1aR*) is expressed by a small population of POA GnRH neurones 63 and blockade of AVP receptors attenuates LH and PRL release 64. In SCN‐lesioned animals, time‐of‐day‐dependent AVP administration in the medial preoptic area rescues the LH surge 65, 66, 67. In the CLOCK‐Δ19 mutant mouse, where the activity of the circadian clock is severely disrupted, and the LH surge is dampened, the expression of AVP and *V1aR* in the SCN is also significantly reduced 68. Administration of AVP into the POA of these animals rescues the LH surge 68.

In the past few decades, it has also become increasingly clear that electrical and TTFL activity of SCN neurones contributes to the regulation of seasonal rhythms 69. Day‐length encoding by the SCN relies strongly on the plasticity of its neuronal network 70, which allows this brain structure to shorten and lengthen the phase relationship among its neurones, coding for the short winter and long summer days, respectively. This regulates the activity of key regions of the body that are important for seasonal changes in reproductive function, such as the seasonal release profile of the pineal gland neurohormone melatonin 69.

Taken together, these observations link the activity of the SCN, the plasticity of its neural circuits and the rhythmic release of some of its neuropeptides with the fundamental control of neurohormone secretion.

## Extra‐SCN endocrine circadian clocks

The discovery and understanding of the inner workings of the TTFL come with the realisation that, although the SCN harbours the principal daily clock, many areas of the brain and body also have local circadian clockworks 71, 72. These act in concert with the SCN to give rise to the extended circadian system. Indeed, core circadian clock genes are active in several neuroendocrine regions of the brain, pituitary and pineal glands 73, 74, 75, 76, as well as in a number of peripheral endocrine tissues, such as the adrenal gland, testis and ovary 77, 78. At the single cell level, clock gene expression is detected in GnRH and neuroendocrine dopaminergic neurones 79, 80.

Furthermore, the daily expression profile of key clock genes, such as *Per1*, reveals rhythmic activity in several neuroendocrine brain regions and individual neurones, such as in dopaminergic cells that control PRL secretion 79, 81, 82, 83. Studies using pituitary explants from transgenic animals bearing bioluminescent reporters of circadian clock activity have conclusively shown that pituicytes can sustain intrinsic circadian rhythms in clock gene expression persisting over several days 84, 85. Rhythmic expression of core clock genes is also reported in the pineal gland 86, in hypothalamic GnRH neurones 87, 88, 89 and in immortalised GnRH cell lines 80, 87, where they serve to regulate GnRH cell activity and gate the sensitivity of these cells to daily hormonal signals 90, 91. Disruption of clock gene activity in GnRH cell lines interrupts daily GnRH release in these cells 91 and, in preoptic‐explants from *Bmal1* knockout mice, the stimulating drive in GnRH release is significantly compromised 88. Clock gene mutant rodent models are now confirming that disruption of appropriate circadian timing signals impairs hypothalamic and peripheral hormone secretion and interferes with reproductive success 92, 93.

Taken together, these observations demonstrate that the functioning of local circadian clocks in neuroendocrine tissues and at the level of the SCN is important for normal neuroendocrine function. These neuroendocrine clock circuits may not only serve as gating mechanisms that determine whether and how neuroendocrine cells respond to periodic SCN time cues, but also provide tissue‐specific local circadian time signals. This may influence output feedback signals from these tissues to the SCN 94. As such, the extended circadian system interacts to maintain optimal daily temporal alignment in physiology across the many tissues and organ systems of the body, although how SCN output molecules affect circadian gene expression (e.g. *Per1*) in neuroendocrine cells remains unknown.

## Neuroendocrine feedback to the SCN: neuroendocrine neurones talking back

Although there is limited evidence of direct neuroendocrine cell projections to the SCN 95, 96, 97, 98, several rhythmic hormonal output signals originating from neuroendocrine cell populations are known to act in the SCN. For example, in all animal species studied thus far, including humans, high‐affinity receptors for melatonin, oestrogen, androgen and progesterone are present in the SCN 99, 100, 101, 102, 103, 104. These hormones can act to modulate the electrical activity of SCN neurones and adjust the phase of the SCN clockworks 105, 106, 107, 108, 109. Furthermore, enzymatic conversion of these hormones, such as the aromatisation of testosterone to oestrogen, provides additional complexity to hormonal signals conveyed to the SCN 110. However, unlike the well‐studied actions of feedback signals (e.g. NPY, serotonin and melatonin) on SCN clock functions, it remains unknown whether the phase of the molecular clockwork and the resulting electrical state of SCN neurones determines whether and how sex hormones act in the SCN. Moreover, how neuroendocrine feedback signals affect SCN circadian gene expression is also not known. Thus, more work is needed before the effects of these hormones on the SCN clockwork can be fully determined.

**Figure 3:**
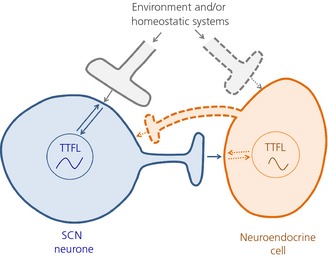
A schematic view of communication between the transcription–translation molecular feedback loop (TTFL) in the suprachiasmatic nucleus (SCN) and neuroendocrine cells through the membrane clock. In this model, signals travelling to and from the TTFL both in the SCN and neuroendocrine clock neurones must flow through the cell membrane (bidirectional arrows) by unknown mechanisms. This process may underlie how the SCN TTFL outputs circadian signals to neuroendocrine cells (blue solid axon and arrow) and how neuroendocrine TTFL feedback to adjust circadian timing in SCN neurones (orange dotted axon and arrow). Through similar unknown processes, environmental and/or homeostatic cues can sculpt the activity of the TTFL in SCN and endocrine neurones (grey solid and dotted axon terminals, and arrows). Solid and broken axons/arrows show known and tentative links, respectively.

Although significant recent progress in circadian neuroendocrine studies now places the activity of the newly‐identified kisspeptin and RFamide‐related peptide at the core of the communication conduits interfacing SCN signals with neuroendocrine activity 111, 112, many key questions still remain. For example, how does circadian information encoded by the hands of the clock (TTFL) translate into meaningful outputs that can be processed by the rest of the brain, including neuroendocrine cells, and how does the brain talk back to sculpt circadian timing in SCN clock neurones? Furthermore, retinal melanopsin neurones that project to the SCN via the RHT also form functional connectivity with a number of neuroendocrine hypothalamic regions 113, 114. Therefore, can environmental light directly modulate the activity of hypothalamic neuroendocrine clock neurones and influence circadian and seasonal timing in these cells? To achieve such communication, signals travelling to and from the TTFL both in the SCN and neuroendocrine clock neurones must flow through the cell membrane (Fig. 3). However, the mechanistic nature of these signals that interweave the molecular clockwork and membrane activity to produce physiological circadian rhythms is largely unknown. This is a dilemma that is now hampering major progress in circadian biology research, although some progress has been made in the SCN. Below, reference is made to the membrane clock as a model for addressing the mechanisms of information flow between the membrane and TTFLs: the all‐important electrical–genetic interplay.

## The membrane clock

Over the past 10 years, pioneering studies have revealed that the membrane properties, activity and excitability of SCN neurones are not only the proximal target of the TTFL, but also act to transmit information from the external world and body to the circadian molecular clockwork (Figs 2 and 3). Indeed, work on mammalian and *Drosophila* clock neurones strongly supports the concept that the electrical activity of clock neurones is integral to the functioning of the intracellular molecular clockwork 115, 116, 117, 118, 119. Indeed, the electrical state of clock neurones can impose time‐of‐day stamps onto its transcriptional programmes, thereby acting as an intrinsic zeitgeber (time‐giver) 120. Studies performed in cultured neonatal SCN slices have also established a tight link between the TTFL activity, as measured by bioluminescence imaging, and membrane excitability in clock neurones. Here, abolishing AP discharge by tetrodotoxin (TTX) blockade of sodium channel activity, which also stops AP‐dependent neurone‐to‐neurone communication, desynchronised the SCN clock and dampened TTFL rhythm amplitude within individual SCN neurones 121, 122. Interestingly, however, similar analysis using SCN brain slices from young adults showed that TTX administration had much less dramatic effects (123; A. T. Hughes and H. D. Piggins, unpublished observations). In support, *in vivo* chronic TTX infusion in adult animals also failed to interfere with SCN function 124. This highlights the complex relationship between the TTFL and membrane clock and/or suggests developmental issues within the SCN neuronal network that require careful consideration 125. Therefore, from the paracrine influence to the electrical–genetic interplay in single SCN neurones, the SCN neural network plays a critical role in adult SCN function 70.

Nevertheless, growing evidence supports the idea that information flow between the membrane and TTFL may well underlie the processes by which neurotransmitters that are released from retinal cells and the body's homeostatic systems sculpt the timing precision in the SCN. TTFL–membrane communication might also underpin how the SCN network organises excitability of its neurones to accommodate extrinsic cues to communicate circadian and seasonal signals to extra‐SCN TTFLs in the body (Fig. 3). Our limited mechanistic understanding of how this is achieved, however, may be inherently linked with the way we measure and report excitability in clock neurones, primarily in the SCN.

## Further considerations: measuring electrical excitability in SCN neurones

Although the rate at which SCN neurones discharge APs is routinely used to report clock phase and functionality, measurement of AP firing frequency alone is not providing us with the complete picture. This is because SCN neurones also show overt day–night differences in their RMP and input resistance (Fig. 1 126, which are other important modes of excitability in SCN neurones. Relying on AP firing rate measurements alone means that excitability in SCN neurones cannot be assessed when they are not producing APs; even in the up state during the day, single SCN neurones generate APs for 4–6 h only and not for the duration of the light phase. Further complexity arises when considering that not all SCN neurones contain a functional molecular clock 70.

Indeed, targeted recording of fluorescence tagged SCN neurones [from mice in which enhanced green fluorescent protein (EGFP) indicates *Per1* promoter activity (*Per1*‐EGFP+ve cells)] 127 demonstrates that the electrical activity of SCN neurones is more complex and richer than was first assumed 128. It was revealed that the well‐described day–night difference in AP generation in the SCN mostly comprises the activity of neurones in which the EGFP construct cannot be detected (EGFP‐ve cells: presumed nonclock neurones). *Per1*‐EGFP+ve SCN neurones stop discharging APs in the middle of the day as their RMP becomes too positive (~ −30 mV) to sustain AP generation. Instead, through reducing the activity of their calcium‐activated potassium channels, these neurones enter a state of intrinsic depolarisation silencing (depolarisation blockade), producing L‐type calcium channel dependent depolarised low‐amplitude membrane oscillations (DLAMOs) instead of APs 128 (Fig. 1 b). These modes of excitability may be important for the normal functioning of the circadian clock, and may be necessary to modulate intracellular calcium levels in SCN neurones, a key signalling molecule in SCN circadian rhythm generation processes 3, 4. Indeed, the relative inability of TTX to suppress TTFL rhythms in young adult SCN neurones 123 suggests that continuous AP production may not be the sole mechanism for intercellular communication and calcium signalling in this brain structure.

In recent years, several studies using genetically encoded calcium sensors (e.g. GCaMP3) or synthetic fluorescent probes (e.g. Fura‐2) have documented the steady‐state intracellular calcium [Ca^2+^]_i_ concentration in SCN neurones. Most 129, 130, 131, 132, but not all 133, reported higher [Ca^2+^]_i_ levels during the day than at night. Furthermore, the concentration of [Ca^2+^]_i_ reported by these studies across the day and at night is not consistent. This ranges from 191 to 440 nm during the day and from 50 to 119 nm at night. Moreover, although, under some experimental conditions, TTX abolished the day–night difference in [Ca^2+^]_i_ levels 134, in others, TTX had no effect 131. Although, across experiments, animal age (neonate versus young adult tissues), species differences (rat versus mouse) and [Ca^2+^]_i_ detection methods (variety of genetically encoded calcium sensors used and Fura‐2) may account for some of these discrepancies, there is no doubt that heterogeneity in SCN neurones and as yet unconfirmed excitability states of SCN cells (AP versus TTX insensitive depolarised DLAMOs) contribute significantly to these apparent inconsistencies. In support, from a single study by Hong *et al*. 135, TTX suppression of day–night [Ca^2+^]i rhythms was seen in only half of SCN neurones, leaving daily cycles of this neuronal excitability measure unaffected in others.

Although the steady‐state [Ca^2+^]_i_ concentration induced by DLAMOs is yet to be described, this electrical state may be crucial for optimal daytime calcium signalling and circadian rhythm generation by clock genes 115, 119. Indeed, the predicted [Ca^2+^]_i_ concentration during DLAMOs 115 is consistent with the higher [Ca^2+^]_i_ concentration and calcium channel conductance values [440 nm 131 and > 40 pA 136, respectively] measured experimentally in SCN neurones. An extreme DLAMO‐like depolarised state (~ −30 mV) is observed in cerebellar and habenular neurones 137, 138, 139 and can be induced in central amygdala neurones 140. Although we have yet to determine whether single neurones in these brain areas are cell‐autonomous circadian oscillators, clock genes are expressed in these brain regions 71, 81, 141.

Taken together, these observations show that electrical signalling in the SCN is complex and that extreme depolarisation in central neurones extends beyond the borders of the SCN. This raises the possibility that these severe depolarised electrical states are more widespread than previously assumed and may be necessary for normal brain function. Studying these electrical states may reconcile some of the apparent inconsistencies seen in SCN neurophysiology and may extend our understanding of the all‐important electrical–genetic interactions in this brain structure, which may also highlight functionality in other brain circuits containing circadian clock genes. Indeed, to date, we have no records of day–night electrical excitability and [Ca^2+^]_i_ levels in neuroendocrine neurones. We also have little understanding of whether and how neuroendocrine cells change their excitability to SCN and other hormonal cues over the circadian day, and whether changes in excitability of these cells can influence their genetic programmes (Fig. 3).

## Conclusions and perspectives

Despite our growing understanding of the cell‐autonomous processes that drive circadian rhythms in clock gene expression, our knowledge of how the molecular clockwork interacts with the membrane to drive daily changes in excitability of SCN neurones is limited. Furthermore, the nature of membrane feedback signals that convey external information to the SCN molecular clockwork is also unknown. Clock genes are found in many of the body's tissue and organ systems, and these extra‐SCN clocks are considered to be vital in providing local circadian timing in a tissue‐specific manner 142. Understanding the nature of the signals responsible for reciprocal connectivity between the molecular clockwork and membrane activity will unravel how circadian rhythms are generated and communicated in SCN and endocrine neurones. Furthermore, this will also reveal how circadian gene activity in neuroendocrine cells gates excitability in these cells to hormones, SCN signals and other physiological demands. This is important if we are to understand how circadian signals influence reproductive neuroendocrinology.

The observation that SCN neurones can convey circadian time cues to each other and to the body both by neural and paracrine signals demonstrates the diversity and richness of communication ‘modes’ in the SCN. Targeted recording of SCN neurones is also adding to our understanding that the electrical repertoire of SCN neurones is much broader than first appreciated. This highlights the challenges facing chronobiologists, physiologists, endocrinologists and neuroscientists in understanding circadian rhythm generation and communication in both the SCN and its targets. It is therefore prudent that, in addition to AP frequency measurements, multiple parameters of membrane excitability are assessed, preferably simultaneously, when interrogating the electrical state of SCN and extra‐SCN neurones. This will greatly increase our understanding of the processes governing cross‐talk between the master circadian clock and the neuroendocrine system, and how these signalling conduits are influenced by external cues.
